# Catchment to coast contaminant transport to a protected marine park on the East coast of Australia

**DOI:** 10.1007/s11356-026-38162-4

**Published:** 2026-08-24

**Authors:** Melanie Taylor, Daniel Matthews, Benjamin Mos, Symon Dworjanyn, Christian J. Sanders

**Affiliations:** 1 National Marine Science Centre, Southern Cross University, Coffs Harbour, Australia; 2 The University of Queensland, Moreton Bay Research Station (MBRS), School of the Environment, Dunwich, 4183 Australia; 3 Centre for Marine Science (CMS), The University of Queensland, Brisbane, 4072 Australia

**Keywords:** Pesticides, Groundwater, Metals, Estuaries, Pollution

## Abstract

Coastal contamination is an ongoing global concern where metal, nutrient and agrochemical runoff are enhanced by human development along coasts and in catchments. Identification of contaminant mobilisation and transportation behaviours can provide valuable insights into pollutant sources in regions impacted by multi-source pollution disturbance scenarios. Because there are substantial knowledge gaps surrounding contaminant behaviour in coastal regions impacted by geogenic and anthropogenic contaminants, we investigated contaminant profiles and transport pathways at five freshwater sites, two coastal lagoons, two groundwater wells, five beach locations and four sites along a marine transect within a developing coastal catchment. The focus of this study was on contaminant delivery to an adjacent marine park located on the East Coast of Australia. In this study, four pesticides were identified over the research period, including imidacloprid, diuron and chlorantraniliprole, during the high flow events in 2022, and tebuconazole, during low flow periods in 2023. This study also identified zinc (up to 267 µg L^−1^) and copper (up to 2.46 µg L^−1^), as well as total nitrogen (up to 744 µg L^−1^) and phosphates (to 163 µg L^−1^) to be exceeding local water quality guidelines at multiple locations within the catchment. Copper fluxes were found to surpass guidelines during high rainfall events, whereas zinc remained above guidelines in 86% of sampling regimes along the catchment, in groundwater, and the coastal ocean. Pollutant levels suggest that pesticides primarily originate from horticultural industries, while metal and nutrient contaminants likely arise from a combination of geogenic and anthropogenic inputs. The relative contribution of each source varies with water flow conditions and interannual variability. The results found here suggest that multiple inputs should be considered when determining sources and contaminant transport pathways to the coastal ocean.

**Graphical Abstract:**

**Figure:**
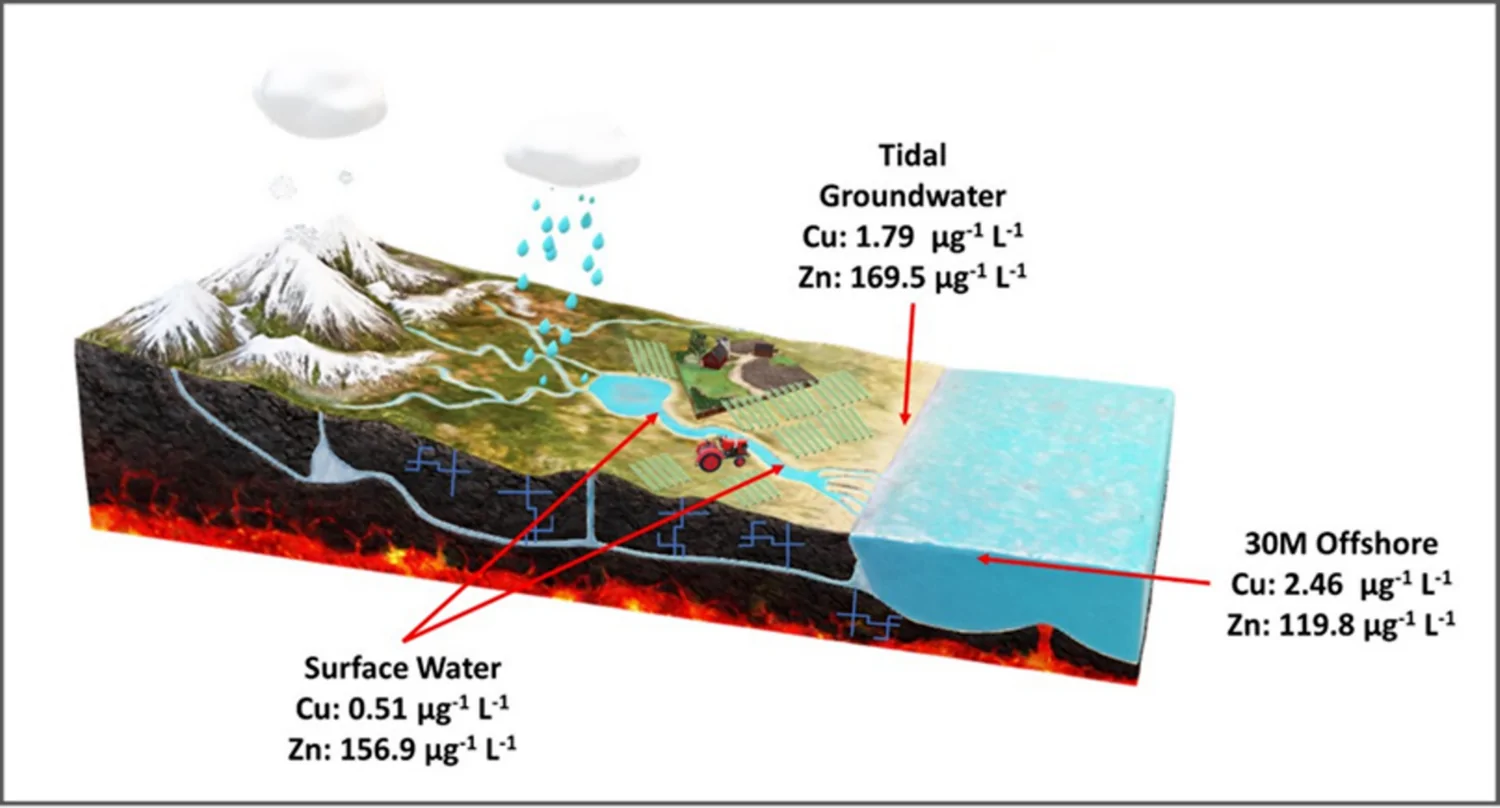

## Introduction

Anthropogenic contamination of coastal aquatic and marine ecosystems is an ongoing environmental concern globally which largely results from agricultural, industrial and residential development along coasts and within catchments (Lu et al. 2018). However, not all aquatic contamination is caused by anthropogenic activities. Aquatic contamination can also be a result of natural processes (Ismanto et al. 2023; Kapoor and Singh 2021). For example, geological drivers of metal contaminants include volcanic activity, as well as the weathering of metal bearing rock formations (Kapoor and Singh 2021). In geologically metal rich catchments, the interplay between natural geogenic fluxes and anthropogenic contamination is not yet fully understood, especially in relation to their combined influence on sensitive marine ecosystems.

Contemporary land use practices have resulted in nutrient, metals and agrochemical runoff to the coastal ocean on a global scale (Henriques et al. 2015; Moreira et al. 2023). Agrochemicals are applied at a rate of 2 million tons per annum worldwide, with fungicides accounting for 17.5%, insecticides approximately 29.5% and herbicides 47.5% of chemical application (Boretti and Rosa 2019). Pesticides and fertilisers have been directly linked to anthropogenic nitrogen contamination in aquatic ecosystems (Boretti and Rosa 2019), with nutrient loading predicted to increase over coming decades in response to increased demand for food resources (Boretti and Rosa 2019). This type of nutrient contamination can result in eutrophication, impacting biogeochemical cycles and ecosystem services (Wadnerkar et al. 2019)

In addition, aquatic ecosystems influenced by agricultural runoff may become exposed to potentially toxic metals and metalloids, such as arsenic (As), mercury (Hg), copper (Cu) and zinc (Zn) (Real et al. 2024), as a result of these substances being used in specific pesticides or the remobilisation of resuspended soils (Conrad et al. 2021). High background metal contents combined with anthropogenic inputs can push concentrations of metals in runoff water over specific environmental trigger values (Hao et al. 2024; Qin et al. 2022). Pollutant runoff to the aquatic environment can introduce toxic compounds that cause stress and mortality in marine species (Boretti and Rosa 2019; Henriques et al. 2015; Laicher et al. 2022). Some contaminants are toxic to biota across trophic levels (Michalak and Messyasz 2021), impacting microorganisms (DeLorenzo et al. 2001; Li et al. 2020; Yin et al. 2015), invertebrates (Handy 1994), fish (Rubalingeswari et al. 2021) and aquatic mammals (Marcovecchio et al. 1994; Reijnders et al. 2009). The contamination of aquatic ecosystems, and the toxic effects of pollutants on resident biota, is a key factor driving the decline in biodiversity across the planet (Boretti and Rosa 2019).

Coastal groundwater can also act as a pathway for contaminant mobilisation to the broader marine environment through submarine groundwater discharge (Li et al. 2004; Zhang et al. 2002). Anthropogenic pollutants originating from mining, agricultural, urban and manufacturing activities can percolate down to the groundwater interface (Moody 1990; Power and Schepers 1989). In regions with geologically high metals background, the dissolution of metals from surrounding bedrock can occur, affecting the concentration of these substances along the catchment and to the coastal ocean (Hao et al. 2024). The identification of contaminant mobilisation and transportation behaviours can provide a valuable insight into pollutant sources (Nordstrom 2011).

Coastal ecosystems provide habitat, nurseries and trophic resources to many marine species (Laicher et al. 2022). Coastal regions are particularly at risk to the impact of pollutants as they act as repositories for contaminants from entire catchments, hence are particularly susceptible to the toxicological risks associated with aquatic pollution (DeLorenzo et al. 2001). Further, pollutants can be mobilised in dissolved forms within the water column or bind to organic matter and fine particles, and transported to the broader marine environment (Conrad et al. 2021). The presence of dissolved and sediment-bound pollutants has the potential to influence biogeochemical processes that occur in coastal ecosystems, such as nutrient cycling (Harley et al. 2015) and carbon fixation (Woodwell et al. 1973). Understanding pollutant dynamics within catchments is an essential step in tracing pollutants through filtration, sequestration, and transformation processes and subsequent mobilisation to the coastal ocean (Conrad et al. 2019), but to date, few studies have explored pollutant dynamics in the context of high geological background metal and/or nutrient levels exacerbated by anthropogenic inputs.

Given the knowledge gaps surrounding contaminant behaviour in coastal regions impacted by geogenic and anthropogenic contaminants, we investigate contaminant profiles, transport pathways and hydrological controls in a developing coastal catchment, focusing on their delivery to an adjacent marine park. Discreet sampling of metal, pesticide and nutrient contaminants, in multiple catchment locations, was conducted to identify mobilisation and transportation behaviours. We build on the literature by (1) identifying contaminant mobilisation behaviours in two sub-catchments impacted by multi-source contamination scenarios, (2) assessing contaminant transportation pathways to the coastal ocean and (3) detecting geological drivers of aquatic contaminants to sensitive marine environments. We hypothesise that in geologically metal-rich catchments, both geogenic and anthropogenic sources may contribute to high concentrations of metal fluxes to the coastal ocean.

## Methods

### Site introduction

**Fig. 1:**
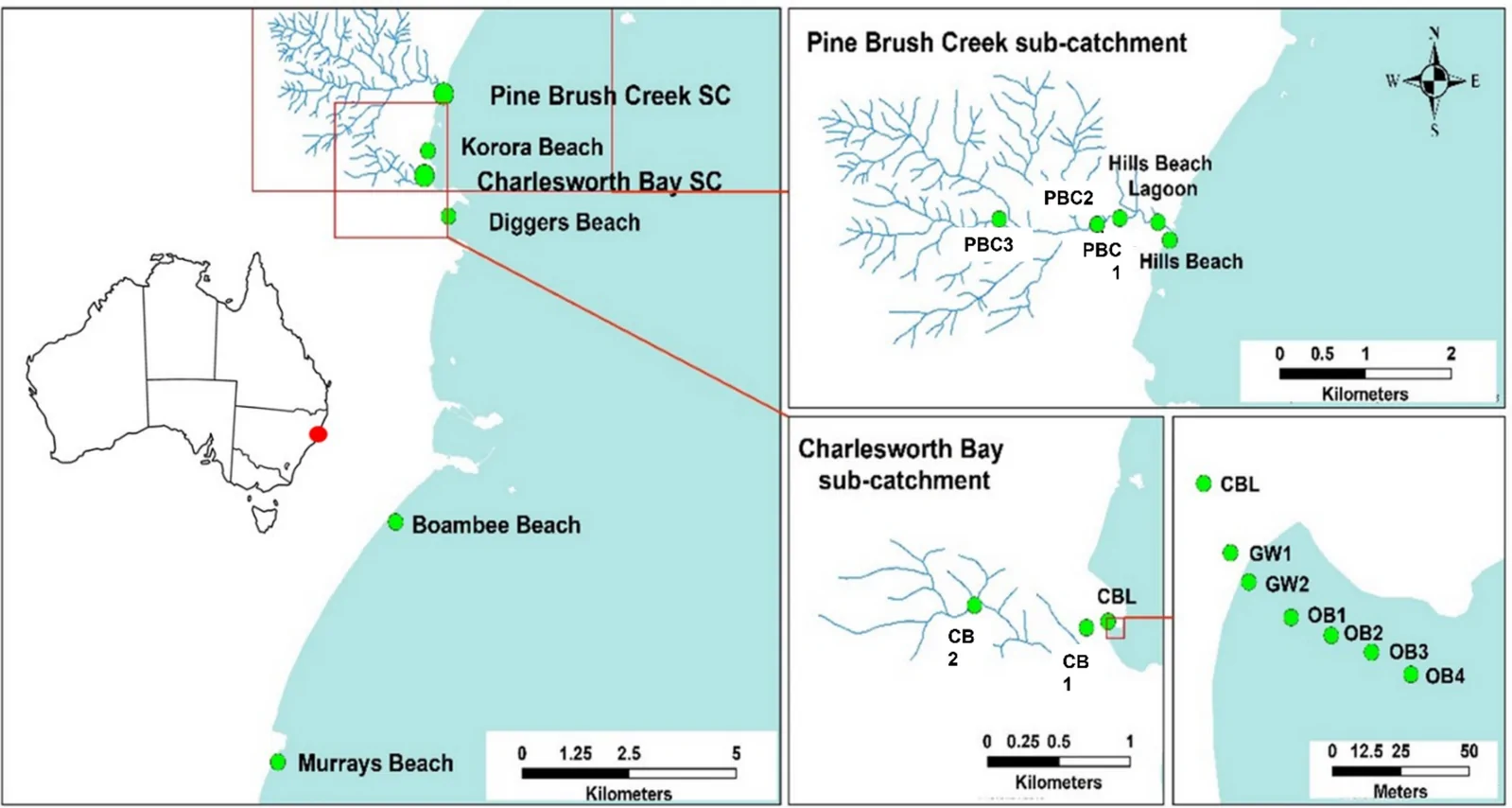
Location of the study sites in this work on the East Coast of Australia. These locations include beach sites; Pine Brush Creek sub-catchment sites (PBC1, PBC2, PBC3, Hills Beach lagoon (HBL) and Hills Beach (HBB)); Charlesworth Bay sub-catchment sites (CB1, CB22, Charlesworth Bay Lagoon (CBL) and groundwater and ocean transect sites (GW1, GW2, OB1 (10 m), OB2 (20 m), OB3 (30 m), and OB4 (40 m)) and surrounding beach locations including the following: Korora Beach (KB), Diggers Beach (DB), Boambee Beach (BB) and Murrays Beach (MB)

The research for this study was conducted across two sub-catchments, several coastal lagoons, groundwater wells, beaches and ocean transect within the Solitary Islands Marine Park (SIMP) (see Fig. 1 for the location of each of the study sites). Five sites across two sub-catchment systems were selected for monitoring where they were subject to 5-day sampling regimes. The Pine Brush Creek system is comprised of PBC1 (lower catchment), PBC2 (mid catchment), and PBC3 (upper catchment) (Fig. 1). Land used for agriculture and urban development surrounds this system. The Charlesworth Bay system hosts CB1 (lower catchment) and CB2 (upper catchment). The CB1 site is located on a man-made lake situated within a golf course. Two groundwater wells (GW1 and GW2); two lagoons (CBL and PBCL); four marine sites situated 10, 20, 30 and 40 m offshore (OB1, OB2, OB3 and OB4); as well as five beach locations were selected for sampling (Fig. 1). Sampling was conducted during the peak of seasonal agricultural activity; at the end of spring in November of 2022, and repeated at the start of summer in December of 2023, to identify temporal consistency of contamination behaviours.

The SIMP is one of eight parks within the temperate east marine parks network and is located at the mixing point for the warm East Australian Current and cooler southern waters, thus providing habitat for a variety of unique temperate, sub-tropical and tropical marine biota (Malcolm et al. 2011). The SIMP supports several threatened species including the critically endangered *Carcharias taurus* (grey nurse shark) (Smith et al. 2014) and is the only known site in the world for the macroalgae *Nereia lophocladia* (Yee et al. 2019). The geological features of the research area are unique to this region of the Australian eastern coastline, located on the Coramba Beds of the Coffs Harbour Block within the New England Orogen (NEO) (Fergusson 2010) . The Coramba beds are comprised of felsic volcanics, metabasalt, lithofeldspathic wacke, chert, jasper, minor siltstone, siliceous siltstone, calcareous siltstone and mudstone (Gilligan et al. 1992). The NEO began formation in the early Cambrian Period of the Palaeozoic Era by compressional and extensional events (Jessop et al. 2019). A volcanic arc, active up until the end of the Carboniferous Period, heavily influenced geological features of the region (Champion et al. 2016; Jessop et al. 2019) as did rifting events post volcanic disturbance (Jessop et al. 2019).

Mineral deposition can be seen to closely follow the migration of volcanic activity (Gilligan et al. 1992). For example, gold occurrences have been reported across the Coffs Harbour and surrounding regions (Gilligan et al. 1992) and historically were the focus of small-scale mining activities. Zinc, arsenic, copper and mercury deposits have also been identified (Gilligan et al. 1992). Soil types as defined by the Australian Soil Classification (ASC) system are predominantly kandosols, with rudosols occurring along the coastline. Acidic kurosols are prominent in the catchment; these soils are associated with high sodium, magnesium and/or aluminium geochemical profiles (Isbell 2016). In neighbouring catchments, iron and organic-aluminium associated podosols are dominant.

Aquifers in the study region are extensive but exhibit low to moderate productivity, being fractured and fissured within the bedrock (Geosciences Australia 2023). Partial mapping of the coastal seabed across the SIMP identified platform and patch reefs, high-intensity ridges, peaks, slopes and low to high-intensity channels (Linklater et al. 2019). Submarine canyons are extensive along the continental shelf in this region. The annual precipitation in the study region is approximately 1700 mm, with episodic rainfall influencing coastal catchment hydrology (Wadnerkar et al. 2019). Previous research within the SIMP has reported a range of aquatic contamination issues, including pesticides (Laicher et al. 2022), metals (Conrad et al. 2019), greenhouse gas emissions and nutrient pollutants (Wadnerkar et al. 2019).

### Sample collection

#### Field parameters

Environmental parameters were recorded at sites selected for discrete sampling over a 5-day period in late November 2022 and again in early December 2023. Temperature, pH, dissolved oxygen, oxidation reduction potential (ORP) and conductivity of surface water were recorded in triplicate from each sampling location (Fig. 1), using a Hach HQ4300 multi-meter calibrated to industry standards (Laffont-Schwob et al. 2015; Rybak et al. 2012). Water samples were collected in high-density polyethylene (HDPE) bottles 30 cm from the surface, or at mid-depth in shallow systems, at the same time daily for 5 consecutive days at the Charlesworth Bay (CB1, CB2) and Pine Brush Creek (PBC1, PBC2, PBC3) sub-catchment sites, and once at lagoon (KBL, CBL), groundwater well (GW1, GW2), ocean transect (OB1, OB2, OB3, OB4), and beach locations (MB, BB, DB, KB). A cross section was taken from sub-catchment sites and flow determined utilising the float method; measurements were repeated during each discrete sampling event to overcome uncertainty in recorded values (Dobriyal et al. 2017). Sample treatment specific to nutrient, pesticide and metal analysis are described in relevant subsections below.

#### Groundwater

Radium sampling took place along a transect, from beach wells to offshore waters during low tide, in each respective year. Water samples were collected at six sites, from beach and to 40 m offshore in 2022 and 2023 (Fig. 1). Offshore samples were collected into 100-L containers, while groundwater samples were collected by digging wells along the sandy beach. In situ, samples were pumped through a canister containing MnO_2_-coated fibre filters that adsorb the radium radioisotopes (^223^Ra and ^224^Ra). Fibres were removed from the canisters and placed in an air-tight Ziploc bag and transported to the laboratory. In the laboratory, the fibres were washed with radium-free water to remove salt and sediment particles. A Radium Delayed Coincidence Counter (RaDeCC) system was used to measure the activity of ^223^Ra and ^224^Ra in each sample on the same day samples were collected (Gomez-Alvarez et al. 2019). The analytical uncertainty is a function of activities encountered, volume sampled and counting time.

#### Nutrients

Water samples for nutrient measurements were passed through 0.45 µm pore size microbiological filters into 10 mL polyethylene vials, transported on ice and stored at −20 °C. Total dissolved nitrogen (TDN), ammonium (NH_4_^+^) and nitrate (nitrite (NO_x_) were then determined via Lachat Flow Injection Analyser as per APHA 4500 (Wadnerkar et al. 2019). Dissolved organic carbon (DOC) samples were passed through 0.7 µm filters into borosilicate vials and processed to APHA 2017 standard.

#### Metals

Water samples for dissolved metals analysis were passed through 0.45-μm microbiological filters and treated with 0.1 mL of nitric acid (HNO_3_) (15.8 mol L^−1^) (Conrad et al. 2021). Samples were transported on ice to laboratory and stored at −6 °C. Samples were digested in 1:3 HNO:HCl and dissolved fraction of arsenic (As), aluminium (Al), copper (Cu), manganese (Mn), barium (Ba) and zinc (Zn), and background salt concentrations were identified using ICP Mass Spectrometry (ICP-MS) calibrated to industry standard APHA 3125 (Conrad et al. 2019). Results were analysed against ANZECC freshwater and marine water quality guidelines. The metals selected for this study were based on geogenic background associations and high regional concentrations identified from previous research in this region of Australia.

#### Pesticides

Water samples for pesticide analysis were collected once per sampling period at all catchment, groundwater, lagoon, beach and ocean transect sites (Fig. 1). Samples were passed through 0.45-µm microbiological filters into amber glass bottles, transported on ice and stored at −20 °C to prevent compound degradation (Laicher et al. 2022; Rybak et al. 2012). Samples were analysed via liquid chromatography with tandem mass spectrometry (LC–MS/MS) as per NEPM 2013 B3 standards for the presence of 173 pesticidal compounds. Sampling occurred in late November 2022 and was repeated in early December 2023.

#### Quality control

Each analytical batch was analysed by laboratories accredited by National Association of Testing Authorities (NATA) under ISO/IEC 17025 and included method blanks, instrument blanks, matrix spikes, laboratory control samples and duplicate analyses; internal standard recoveries and matrix spike recoveries were used to assess extraction and instrument performance. Multi-point calibration curves (minimum five points; *r*^2^ ≥ 0.99) from traceable certified standards were used for quantification, and calibration verification standards were analysed regularly throughout each sequence. For ICP-MS runs, internal standards were applied to correct for matrix effects and instrument drift. Limits of detection (LOD) and quantification (LOQ) were determined per analyte (LOD = S/N ≥ 3; LOQ = S/N ≥ 10) or by 3 × and 10 × the standard deviation of low-level replicate measurements/blank analyses as appropriate and were reported in results below. Certified reference materials NASS-7, CASS-6 and NMI MX014 (National Measurement Institute, seawater matrix) were analysed as part of quality assurance procedures.

#### Data analyses

Metal and nutrient fluxes (F) were determined utilising the equation: F = Q × C, where Q = flow rate and C = concentration. Data was analysed using the t-statistic where data was limited to two groups of equal size and by ANOVA for multi-group analysis. Post hoc analysis was performed using Tukey’s HSD.

## Results and discussion

### Field parameters

Precipitation was recorded at a total of 47.3 mm over the 2022 and 5.6 mm over the 2023 regime. The upper Charlesworth Bay sub-catchment site, CB2, recorded an average (*n* = 5) flow of 0.015 m^3^ sec^−1^ in 2022 and 0.003 m^3^ sec^−1^ in 2023. The lower sub-catchment site, CB1, is a manmade lake and as such is not subject to significant surface water movement, much of that likely seeping through permeable sands to the ocean. The Pine Brush Creek sub-catchment incurred flows of between 0.038 and 0.145 m^3^ sec^−1^ in 2022 and between 0.0006 and 0.013 m^3^ sec^−1^ in 2023 (see Supplementary Material).

The average temperature during this study ranged between 20 and 24 °C in 2022 and between 24 and 28 °C in 2023. Analysis via ANOVA reported a significant difference in water temperature between sites in both 2022 (*F*(4, 20) = 5.08, *P* = 0.005, *n*^2^_p_ = 0.50. Water temperature at CB1 was significantly higher than comparative sites during both research periods (*P* = < 0.001). Salinity within the Pine Brush Creek sub-catchment was recorded in the order PBC1 > PBC2 > PBC3 over both the 2022 and 2023 research periods, averaging (*n* = 5) between 0.07 and 0.09 ppt in 2022 and between 0.07 and 0.14 ppt in 2023. Salinity at lower and mid-catchment sites (CB1, PBC1 and PBC2) increased significantly (CB1: *t*(4) = −29, *P* =  < 0.001, PBC1: *t*(4) = −2.29, *P* = 0.04, PBC2: *t*(4) = −8.57, *P* =  < 0.001)) in 2023. The proximity of lower catchment sites to the land–ocean interface makes them particularly susceptible to tidal and groundwater inputs (Mokoena et al. 2021; Werner and Lockington 2006). In rainfall events, the flush of freshwater throughout the catchment can dilute salinity levels at the sites most impacted by tidal marine and groundwater exchange (Prieto and Destouni 2005; Werner and Lockington 2006).

The pH across all sites was between 6.4 and 7.5 throughout both sampling regimes, which is consistent with guidelines for sub-tropical freshwater and estuarine ecosystems (ANZECC 2000). In 2022, dissolved oxygen (DO) saturation was between 90 and 96% throughout the Pine Brush Creek system (DK sites) and at the site located in the upper Charlesworth Bay system (CB2). However, in 2023, DO fell to between 42 and 55% at sites located within the Pine Brush Creek sub-catchment, most likely as a result of the higher temperatures and lower flow recorded during that period. The site located in the lower Charlesworth Bay system (CB1) repeatedly reported DO saturation below 75% on sampling occasions during both sampling regimes. Low DO can result in a number of biogeochemical and bioecological transformations, including dissolution from sediments (Atkinson et al. 2007; Liu et al. 2019), which could be important drivers of the contaminants found in this study (see Supplementary Material).

The oxidation–reduction potential (ORP) fell between −20 and 20 at sites within the Korora Basin across the 2022–2023 sampling periods, which is consistent with denitrification processes (YSI Environmental 2008). Sites within the Charlesworth Bay catchment recorded significantly lower (CB1: *t*(4) = 10.9, *P* = < 0.001, CB2: *t*(4) = 5.1, *P* = 0.003) ORP in 2023, with levels indicating the biogeochemical formation of sulfides (YSI Environmental 2008). Increased phosphorus mobilisation has been linked to diminished ORP, as well as sulfide production in coastal hydro systems (Li et al. 2016). Oxidation reduction potential has also been found to influence the solubility of metals (Bourg and Loch 1995; Chuan et al. 1996) which may help to explain why we found high dissolved concentrations of metals in some samples in this study.

For the groundwater results during both years, the radium concentrations were generally higher along the transect than oceanic values (~ 1 dpm 100L^−1^ for ^223^Ra and ~ 17 dpm 100L^−1^ for ^224^Ra (Gomez-Alvarez et al. 2019), indicating groundwater flow to the coastal ocean (Table 1). The concentrations of ^224^Ra and ^223^Ra were higher in 2023. In 2022, concentrations were up to 4.7 and 122.8 for ^223^Ra dpm L^−1^ and ^224^Ra_ex_ along the transect of the beach groundwater samples respectively. In 2023, concentrations were up to 6.4 and 233.4 for ^223^Ra dpm L^−1^ and ^224^Ra_ex_ in the beach groundwater samples, respectively. This high radium concentration in the beach groundwater and coastal ocean is indicative of high groundwater flow to Charlesworth Bay (Gomez-Alvarez et al. 2019). Groundwater is a significant source of nutrients (Tait et al. 2023), metals (Luo et al. 2022; Wang et al. 2022, 2023) and pesticides (Gutiérrez-Martín et al. 2023) to the coastal ocean.

**Table 1:** Radium-223 and ^224^Ra_ex_ in the samples taken during this study

Year	Site	^223^Ra dpm 100L^−1^	^224^Ra_ex_ dpm 100L^−1^
2022	Well 1	4.7	122.8
	Well2	2.4	136.1
	Beach 1	0.8	22.7
	Beach 2	0.7	16.2
	Beach 3	1.1	18.3
	Beach 4	1.1	19.9
2023	Well 1	1.1	31.8
	Well2	6.4	233.2
	Beach 1	1.2	39.2
	Beach 2	2.4	68.8
	Beach 3	5.3	80.1
	Beach 4	0.9	18.9

### Nutrients

**Fig. 2:**
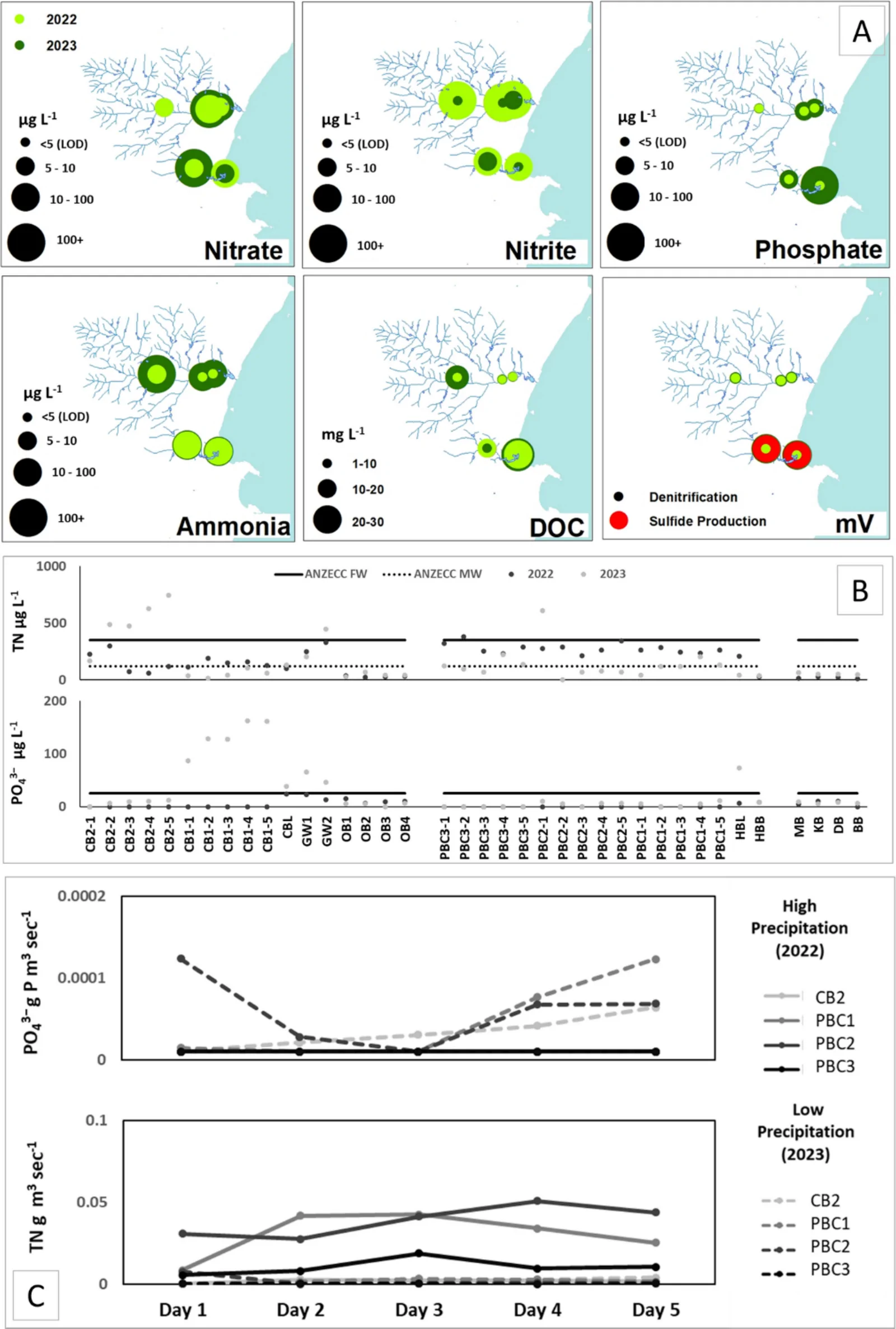
**a** Average (*n* = 5) nutrients and oxidation reduction potential (ORP) at Pine Brush Creek sub-catchment sites (PBC1, PBC2, PBC3) and Charlesworth Bay sub-catchment sites (CB1, CB2), and results of discrete sampling for Hills Beach Lagoon (HBL) and Hills Beach (HBB), Charlesworth Bay Lagoon (CBL), groundwater and ocean transect sites (GW1, GW2, OB1 (10 m), OB2 (20 m), OB3 (30 m) and OB4 (40 m)), and surrounding beach locations including the following: Korora Beach (KB), Diggers Beach (DB), Boambee Beach (BB) and Murrays Beach (MB), with standard error bars. Total nitrogen and phosphate in relation to ANZECC guidelines (**b**) and total nitrogen and phosphate fluxes over 2022 and 2023 sampling regime (**c**). ANZECC guidelines adjusted for freshwater and marine environments

In 2022, dissolved organic carbon (DOC) was between 7 and 15 mg L^−1^ in the Charlesworth Bay sub-catchment (Fig. 2a). In the lower flow periods of 2023, DOC was significantly higher (*t*(9) = −16.64, *P* < 0.001) across the catchment (27–37 mg L^−1^). Highest DOC concentrations were found at the ocean transect sites. In the Pine Brush Creek sub-catchment, DOC ranged between 3 and 12 mg L^−1^ in 2022 and between 12 and 30 mg L^−1^ in 2023. Highest concentrations in both years were recorded in the Hills Beach Lagoon (HBL) (Fig. 2a). DOC was significantly higher (*F*(1,36) = 23.54, *P* < 0.001, *n*^2^_p_ = 0.40) at groundwater and beach locations (14–37 mg L^−1^) compared to surface water sites.

Ammonia (NH_3_) concentrations across the Charlesworth Bay sub-catchment and ocean transects ranged between 5 and 76 µg L^−1^ in 2022, with the highest levels recorded at catchment and groundwater sites in order GW2 > CB1 > CB2 > CBL > GW1 (Fig. 2). Ammonia concentrations were recorded between 18 and 84 µg L^−1^ during the 2023 sampling regime in the order GW1 > CB2 > GW2 > CB1 > CBL. These results indicate groundwater is a prominent pathway for NH_3_ transportation to the coastal marine environment in the Charlesworth Bay sub-catchment during high precipitation events. In the Pine Brush Creek sub-catchment, NH_3_ was recorded between 0 and 17 µg L^−1^ in 2022. Ammonia concentrations were significantly higher (*t*(14) = −4.75, *P* < 0.001) across the sub-catchment during the low flow periods of 2023, recorded between 8 and 124 µg L^−1^, with the highest concentrations found at the upper catchment site PBC3.

In 2022, nitrite (NO_2_) ranged between 15 and 251 µg L^−1^ across creek, lagoon, groundwater and ocean transect sites located within the Charlesworth Bay sub-catchment, with highest concentrations being recorded at the two groundwater wells (GW1 and GW2) (Fig. 2). Nitrite was below detectable limits (LOD < 5 µg L^−1^) at many sites within the Charlesworth Bay sub-catchment in 2023, and overall catchment loading was significantly lower (*t*(9) = 3.81, *P* 0.002) during this period. Average (*n* = 5) NO_2_ concentrations at sites in the Pine Brush Creek sub-catchment (PBC1, PBC2 and PBC3) measured between 254 µg L^−1^ and 288 µg L^−1^ during the 2022 research period. The connecting Hills Beach lagoon (HBL) had NO_2_ concentrations at 198 µg L^−1^, indicating partial denitrification was occurring between the PBC3 and HBL locations. However, in 2023, NO_2_ was below the LOD (5 µg L^−1^) or only trace amounts were detected at these same locations. Mobilisation of NO_2_ occurred across both sub-catchments predominantly during periods of higher precipitation. Conversely, in coastal lagoon and groundwater locations, NO_2_ concentrations increased in low rainfall periods suggesting denitrification of NO_3_^−^. High NO_2_ concentrations found at groundwater well locations (GW1 and GW2) during high flow periods indicate groundwater may be contributing to nitrite transportation to the coastal ocean in this region.

Nitrate (NO_3_^−^) concentrations were between 6 and 38 µg^−1^ L^−1^ throughout the Charlesworth Bay sub-catchment in 2022, where highest concentrations were reported in the order CB1 > CBL > GW1 (Fig. 2). Nitrate was significantly higher (CB2: *t*(4) =  −5.03, *P* 0.003) across the upper catchment in 2023, falling between 120 and 435 µg L^−1^. Highest NO_3_^−^ concentrations in 2023 were recorded in the order CB2 > GW2 > GW1, exceeding ANZECC water quality guidelines of 350 µg L^−1^ at the groundwater well 2 location as well as in 80% of sampling occurrences at the CB2 site. However, at the lower catchment site CB1, NO_3_^−^ significantly higher in 2022 than in 2023 (*t*(4) = 3.24, *P* = 0.02). This suggests NO_3_^−^ was mobilised in the sub-catchment upper reaches during low flow periods and subsequently transported to the lower catchment sites during precipitation events. Nitrate concentrations within the Pine Brush Creek sub-catchment were recorded between 1 and 18 µg L^−1^ in 2022, with highest NO_3_^−^ concentrations reported in order HBL > PBC2 > HBB, indicating NO_3_^−^ mobilisation to the coastal ocean, and subsequent dilution in high rainfall events (Poor and McDonnell 2007). Nitrate concentrations were recorded between 4 and 120 µg L^−1^ throughout the catchment in 2023.

Although total nitrogen (TN) concentrations were higher in the Charlesworth Bay sub-catchment, fluxes to the coastal ocean in rainfall events were significantly higher in the Pine Brush Creek sub-catchment (*F*(1,18) = 12.4, *P* 0.002, *n*^2^_p_ = 0.41) (Fig. 2c). This may be influenced by runoff from agricultural land use in the upper catchment reaches (Conrad et al. 2021), combined with the higher flows associated with the more extensive hydrological system. Average TN concentrations were found to fall in the order GW2 > GW1 > CB2 > CB1 > CBL in 2022 and CB2 > GW2 > GW1 > CBL > CB1 in 2023 for the Charlesworth Bay sub-catchment, and PBC3 > PBC2 > PBC1 > HBL in 2022 and PBC2 > PBC3 > PBC1 > HBL in 2023 in the Pine Brush Creek sub-catchment. These findings indicate that groundwater may be contributing to nitrogen transportation to the coastal ocean, and that the source of nitrogen is likely situated in the upper catchment.

Nitrogen contamination of groundwater and subsequent transport to the coastal ocean has been identified previously in the mid-north coast region of New South Wales, Australia, adjacent to the sites sampled in this study (Reading et al. 2017) and in catchments globally (Morrissy et al. 2021; Santos et al. 2021; Tait et al. 2023). For example, submarine groundwater discharge was a significant driver of nutrients to the Great Barrier Reef, Australia (Tait et al. 2023). Nitrate contamination of coastal groundwater in Laizhou Bay, China, exceeded guidelines in 89% of sampling events (Sheng et al. 2023). Further, a worldwide assessment of submarine groundwater discharge found groundwater nutrient inputs to be comparable with surface water loading to the global ocean (Santos et al. 2021), suggesting the monitoring of groundwater contaminants is as critical to global water security as the management and regulation of surface water contamination.

Phosphate (PO_4_^3−^) was below detectable limits throughout the Charlesworth Bay sub-catchment in 2022. In 2023, concentrations ranged from 8 to 134 µg L^−1^ across catchment and groundwater sites and between 0 and 7 µg L^−1^ across the ocean transect. Highest concentrations were recorded at lower catchment and groundwater sites in the order CB1 > GW1 > GW2 > CBL. In 2022, measurements in the Pine Brush Creek sub-catchment found no trace PO_4_^3−^ at catchment sites (PBC1, PBC2 and PBC3); however, PO_4_^3−^ was found at 73 µg L^−1^ in Hills Beach lagoon (HBL) and 8 µg L^−1^ at Hills Beach (HBB). In 2023, PO_4_^3−^ levels exceeded the ANZECC water quality guidelines of 25 µg L^−1^ at Hills Beach Lagoon, Charlesworth Bay Lagoon, the two groundwater sites, and at CB1 throughout the sampling regime (Fig. 2). Analysis via ANOVA found there to be a significant difference in PO_4_^3−^ concentrations between sites in 2023 (*F*(4,20) = 81.26, *P* < 0.001, *n*^2^_p_ = 0.94). The lower Charlesworth sub-catchment site (CB1) reported significantly higher (CB2: *t*(4) = 10.49, *P* < 0.001, PBC1: *t*(4) = 9.67, *P* < 0.001, PBC2: *t*(4) = 8.88, *P* < 0.001, PBC3: *t*(4) = 9.58, *P* < 0.001) concentrations of PO_4_^3−^ than other research sites.

The CB1 site is located adjacent to developed areas that are subject to regular fertiliser and pesticide applications which may be influencing PO_4_^3−^ at this site. Phosphate mobilisation predominantly occurred during periods of low rainfall and is most likely related to sediment release. In anaerobic sulfide-producing environments, the formation of iron sulfides can result in the release of PO_4_^3−^ from sediment to the surrounding hydro-system (Zhao et al. 2019).

### Metals

Arsenic (As) was recorded at Hills Beach (HBB: 3.5 µg L^−1^), Murrays Beach (MB: 3.3 µg L^−1^), Korora Beach (KB: 3.4 µg L^−1^), Diggers Beach (DB: 3.5 µg L^−1^) and Boambee Beach (BB: 4.1 µg L^−1^) in 2022. The levels of As recorded at beach sites are consistent with higher global background concentrations for marine waters (1–3 µg L^−1^) (Wang et al. 2022, 2023). However, coastal ocean sites reported lower As concentrations during the low flow periods of 2023 (HBB: 1.23 µg L^−1^, MB: 1.28 µg L^−1^, KB: 1.22 µg L^−1^, DB: 1.63 µg L^−1^ and BB: 1.34 µg L^−1^) indicating background concentrations in coastal waters of this region of Australia are between 1.2 and 1.7 µg L^−1^. The Charlesworth Bay sub-catchment reported As concentrations to be between 1.6 and 4.5 µg L^−1^ in the order OB1 > OB2 > OB4 > GW1 > CB1 > OB3 > CBL > GW2 > CB2. Arsenic concentrations in the lower catchment and groundwater sites could potentially indicate additional historical inputs of arsenic-based herbicides from the surrounding developed areas (Cai et al. 2002), which could be increasing As loading to the coastal ocean during high rainfall periods. In the Pine Brush Creek sub-catchment, As was recorded at the upper catchment site (PBC3: 1.01 µg L^−1^), which is consistent with expected background concentrations for freshwater systems (Wang et al. 2022, 2023).

In 2023, As concentrations across the Charlesworth Bay sub-catchment were found to be between 0.71 and 5.74 µg L^−1^. In the Pine Brush Creek sub-catchment, As concentrations ranged between 0.75 and 4.12 µg L^−1^ in the order HBL > PBC3 > HBB > PBC1 > PBC2. There was a significant difference in As concentrations between sites in both the 2022 (*F*(4,20) = 26.6, *P* < 0.001, *n*^2^_p_ = 0.84) and 2023 (*F*(4, 20) = 428.50, *P* < 0.001, *n*^2^_p_ = 0.99) research periods. Arsenic concentrations were significantly higher at the lower sub-catchment site CB1 when compared to sites in both sub-catchments in both 2022 (*P* 0.03–< 0.001) and 2023 (*P* < 0.001).

The mobilisation of As during the low rainfall period of 2023 is likely due to sediment-bound As release to surface water. The presence of phosphate can substantially increase the rate, as well as the amount, of As released from sediments by substituting as an As displacing ligand (Rubinos et al. 2011). Also, the reduction of sulfur species and the dissolution of iron oxyhydroxides can influence As release from the sediment (Keimowitz et al. 2005; O’Day et al. 2004), which is supported by the water quality and nutrient results of this study. However, we do not have enough information to determine the source of this arsenic, though the high values indicate an anthropogenic source.

**Fig. 3:**
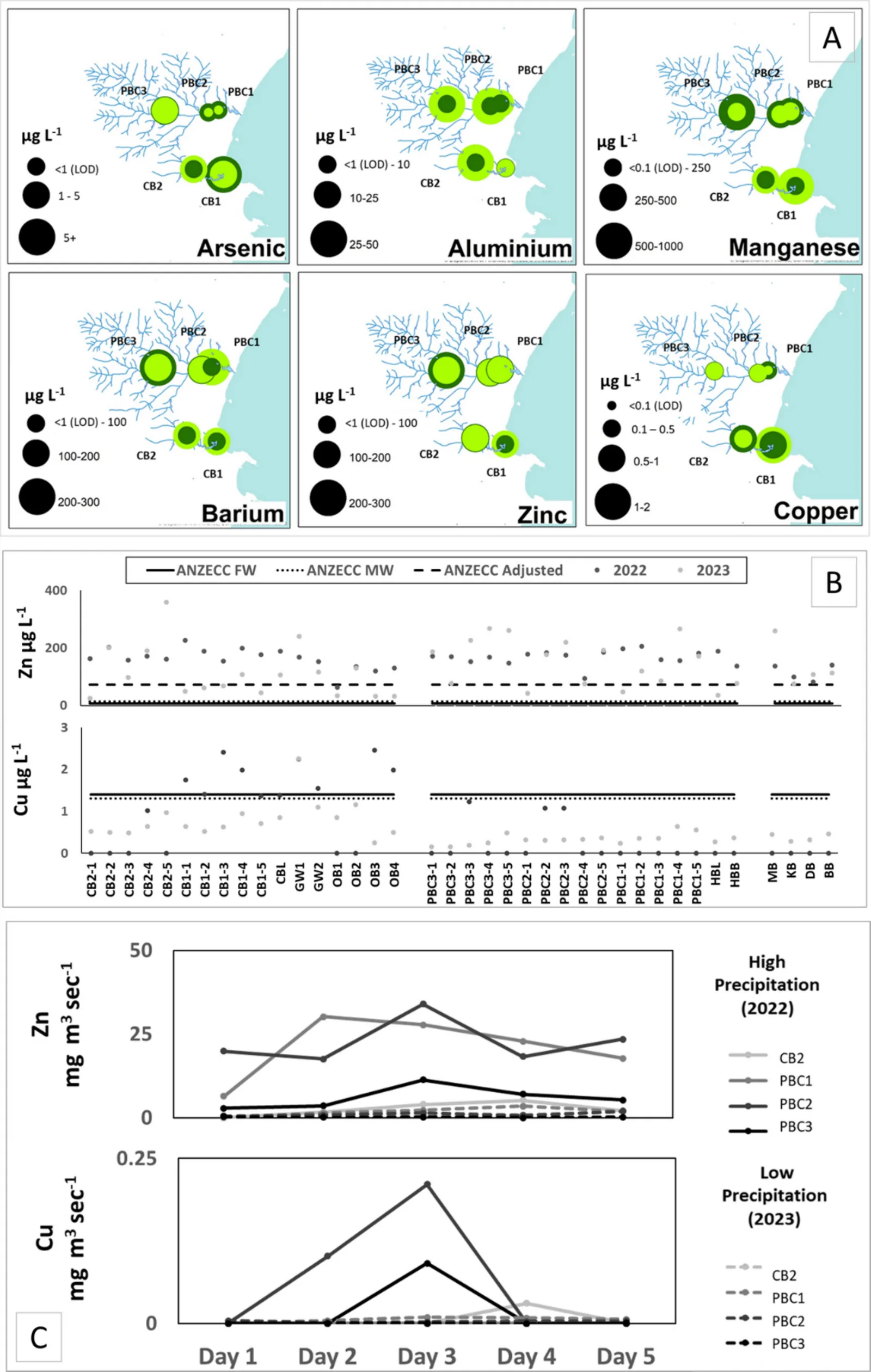
**a** Average (*n* = 5) As, Al, Mn, Ba, Zn and Cu at Pine Brush Creek sub-catchment sites (PBC1, PBC2, PBC3) and Charlesworth Bay sub-catchment sites (CB1, CB2), and results of discrete sampling for Hills Beach lagoon (HBL) and Hills Beach (HBB)), Charlesworth Bay Lagoon (CBL), groundwater and ocean transect sites (GW1, GW2, OB1 (10 m), OB2 (20 m), OB3 (30 m) and OB4 (40 m)), and surrounding beach locations including the following: Korora Beach (KB), Diggers Beach (DB), Boambee Beach (BB) and Murrays Beach (MB), with standard error bars. Zn and Cu in relation to ANZECC guidelines (**b**), and trace metal fluxes over 2022 and 2023 sampling regime (**c**). Note: Trigger values for zinc have been adjusted to maximum conservative values

In the Charlesworth Bay sub-catchment, highest aluminium (Al) concentrations were found to be in the order CB2 > GW2 > CB1 during the 2022 sampling regime. Concentrations were highest at the upper catchment site CB2 (48 µg L^−1^), followed by groundwater well 2 (11 µg L^−1^). Aluminium was reported to be significantly higher (*t*(4) = −2.26, *P* 0.04)) at the upper catchment site when compared to the lower catchment site CB1 (Fig. 3). The Pine Brush Creek sub-catchment recorded Al concentrations between 23 and 37 µg L^−1^ at catchment sites in the order PBC2 > PBC3 > PBC1. Both sub-catchments contain pockets of acidic kurosol soils, which are associated with high Al concentrations (Isbell 2016). No trace Al was reported at lagoon, beach or ocean transect sites, supporting soil associated Al mobilisation in this region.

In 2023, Al concentrations in the Charlesworth Bay sub-catchment were found to be in the order GW2 > CB2 > OB1 > GW1 > OB3 > OB2 > CB1 > CBL > OB4. Aluminium concentrations were recorded at the groundwater well (GW2) location five to ten times higher compared to the concentrations recorded at other catchment sites (26.9 µg L^−1^). Aluminium varied minutely among Pine Brush Creek sites, ranging between 4.37 and 6.86 µg L^−1^. Concentrations recorded at the coastal ocean sites Murrays Beach (MB) and Boambee Beach (BB) were found to be higher than most catchment locations (11 µg L^−1^ and 14 µg L^−1^, respectively). The results of this study indicate that submarine groundwater discharge may be active at these sites, potentially caused by fractures in the bedrock providing a pathway for deep groundwater transportation (Santos et al. 2021). However, the remobilisation of Al contained in marine sediments could also be impacting Al concentrations in these locations (Middag et al. 2015).

Manganese (Mn) was identified predominantly within catchment surface water, with only minor traces detected in groundwater and ocean transect samples (< 5 µg L^−1^). Manganese concentrations in the Charlesworth Bay sub-catchment were found to be significantly higher in 2022 compared to 2023 (CB1: *t*(4) = 2.14 *P* 0.05, CB2: *t*(4) = 7.89, *P* < 0.001) ranging between 422 and 945 µg L^−1^ (Fig. 3). However, the reverse trend is noted in the Pine Brush Creek sub-catchment where Mn concentrations were found to be significantly higher (PBC1: *t*(4) = −6.32, *P* < 0.001, PBC2: *t*(4) = −8.39, *P* < 0.001, PBC3: *t*(4) = −8.89, *P* < 0.001) in 2023 than in 2022, ranging between 294 and 824 µg L^−1^. Manganese silicate ore deposits have been identified within the New England Orogen (Gilligan et al. 1992) and were historically the focus of small-scale mining operations. It is possible disturbance from historical mining efforts have increased Mn availability to surface water in this region.

During the 2022 sampling regime, barium (Ba) concentrations in the Charlesworth Bay sub-catchment recorded in order OB2 > GW2 > OB4 > CBL > OB3 > CB2 > CB1 > GW1 > OB1 and ranged between 38 and 231 µg L^−1^. In 2023, concentrations ranged between 25 and 358 µg L^−1^, and the highest Ba concentrations being found in groundwater locations. In 2023, Ba concentrations in the Pine Brush Creek sub-catchment were recorded in the order PBC3 > PBC2 > PBC1 > HBB > HBL. Barium has been associated with groundwater mobilisation in aquifers around the world (Giménez-Forcada and Vega-Alegre 2015; Marandi et al. 2004; Moore 1997). Barium concentrations at the Hills Beach (148 µg L^−1^), Murrays Beach (169 µg L^−1^), Boambee Beach (197 µg L^−1^) and Charlesworth Bay offshore transect sites (230 µg L^−1^) in 2022 indicate rainfall-driven groundwater exchange had a substantial influence on coastal contaminant transportation in these locations.

Zinc (Zn) concentrations ranged between 63 and 190 µg L^−1^ in 2022 and between 31 and 240 µg L^−1^ in 2023, across the Charlesworth Bay and Pine Brush Creek sub-catchments (Fig. 3). Zinc levels exceeded ANZECC trigger values of 8 µg L^−1^ for 95% species protection at all catchment, groundwater and marine sites throughout the 2022 and 2023 sampling periods (Fig. 3). Zinc bioavailability and toxicity are heavily influenced by calcium carbonate content of the water column (ANZECC 2000), which was not an analysed component of this research; hence, the conservative maximum trigger value as per ANZECC water quality guidelines of 72 µg L^−1^ was applied. Despite using conservative trigger values for freshwater catchment sites, Zn concentrations exceeded guidelines in 86% of sampling events (Fig. 3). Analysis via ANOVA found there to be no significant differences in Zn concentrations between sites in either 2022 (*F*(4,20) = 1.08, *P* 0.39, *n*^2^_p_ = 0.18) or 2023 (*F*(4,20) = 1.86, *P* 0.16, *n*^2^_p_ = 0.27).

Exposure to Zn levels that exceed trigger values is associated with population, reproduction, development, growth and/or mortality impacts for a range of marine species including diatoms (Franklin et al. 2001), green algae (Hooten and Carr 1998), annelids (Gopalakrishnan et al. 2008), anemones (Howe et al. 2014), crustaceans (Ahsanullah and Williams 1991) and mollusks (Martin et al. 1981; Nadella et al. 2009). The high Zn concentrations recorded in this study, most notably at marine protected ocean sites, raise concerns over the toxicological ramifications for biota residing within this ecologically significant region of Australia. Globally, sources of Zn to the environment include naturally occurring mineral deposits (Obiora et al. 2019), as well as anthropogenic inputs from mining, manufacturing, transportation systems and agriculture (Noulas et al. 2018). High fluxes of Zn in rainfall events recorded in this study indicate Zn contamination is likely exacerbated by anthropogenic influences. In this region of Australia, excess Zn may be related to historical land use. Zinc deposits have been identified across a broad regional scale, and remobilisation to surface water and groundwater is most likely responsible for the high background concentrations recorded in this study. The analyses in this research were not sophisticated enough to determine exact source(s) of the Zn. However, the high levels indicate geological and anthropogenic source combinations.

Copper (Cu) concentrations in the Charlesworth Bay sub-catchment were found to exceed ANZECC water quality guidelines of 1.4 µg L^−1^ in 2022 at the lower catchment site CB1, in both groundwater wells GW1 and GW2, as well as the two ocean-transect sites OB3 and OB4, 30 and 40 m offshore. The CB1 site exceeded ANZECC trigger Cu value of 1.4 µg L^−1^ for 95% species protection in freshwater systems during four out of five sampling events in the 2022 sampling period (Fig. 3b). In 2023, Cu was found to decrease significantly at the CB1 (*t*(4) = −5.77, *P* 0.002) and PBC1 (*t*(4) = −5.87, *P* 0.002) locations. Copper concentrations were found to be highest in groundwater sites as well as ocean transect site 2 (OB2) in 2023, though was found to exceed guidelines only at the groundwater well 1 (GW1) location. In the Pine Brush Creek sub-catchment, Cu concentrations ranged between 0 and 0.43 µg L^−1^ in 2022, with highest concentrations recorded in the upper catchment at the PBC2 and PBC3 sites (Fig. 3b). In 2023, Cu concentrations ranged between 0.25 and 0.43 µg L^−1^, in the order PBC1 > HBB > PBC2 > HBL > PBC3. Copper concentrations at the Hills Beach location (HBB) in 2023 were consistent with those recorded at Murrays Beach (MB), Korora Beach (KB), Diggers Beach (DB) and Boambee Beach (BB) sites (0.29–0.46 µg L^−1^).

Copper mobilisation was found to be heavily influenced by precipitation events in this region (Fig. 3b). Highest Cu fluxes were recorded in the Pine Brush Creek catchment at the upper sub-catchment sites PBC2 and PBC3 (0.21 and 0.09 mg m^3^ sec^−1^ respectively) (Fig. 3c). Copper sources in this region include mineral deposits (Gilligan et al. 1992) and a sustained history of agrochemical application (Conrad et al. 2019). The Pine Brush Creek sub-catchment has greater catchment volume and high flows than the Charlesworth Bay sub-catchment and is subject to runoff from intense agriculture in the upper reaches. The stability of Cu concentrations across research locations in periods of low rainfall combined with Cu fluxes to the coastal ocean in rainfall events (Fig. 3c) suggests a multi-source pollution scenario. However, seepage from intense sustained agrochemical application in the upper catchment reaches (Conrad et al. 2019), combined with the fractured nature of the bedrock in this region (Geosciences Australia 2023), has potentially resulted in contaminated groundwater pockets that are subsequently flushed out to the coastal ocean in the form of submarine groundwater discharge in high rainfall events.

Copper can be toxic to aquatic biota at low concentrations, inducing a range of complications to biological functioning, and in extreme scenarios can trigger mortality events (Liao et al. 2023; McKnight et al. 2023). For example, Cu has been found to impact growth and decrease reproductive success rates in fish species (Liao et al. 2023) and inhibit osmotic regulation and impact reproductive function in crustaceans (Banaee et al. 2024). Microalgae appear to be particularly susceptible to Cu toxicity, with several microalgal species experiencing negative effects at concentrations below environmental guidelines (McKnight et al. 2023).

Overall, the results here support the hypothesis that in geologically metal-rich catchments, both geogenic and anthropogenic sources may contribute to high concentrations of metals to the coastal ocean. Persistent high Zn and Cu concentrations indicated multiple sources as indicated by the fluxes during rainfall events. Our findings are consistent with studies in China that found metal sources to be a combination of atmospheric deposition (17.57%), mining (19.38%), agriculture (31.05%) and geological sources (32%), where Cu contaminants were related to mining activities and Zn contaminants found to be derived from geological characteristics (Qin et al. 2022).

### Pesticides

**Fig. 4:**
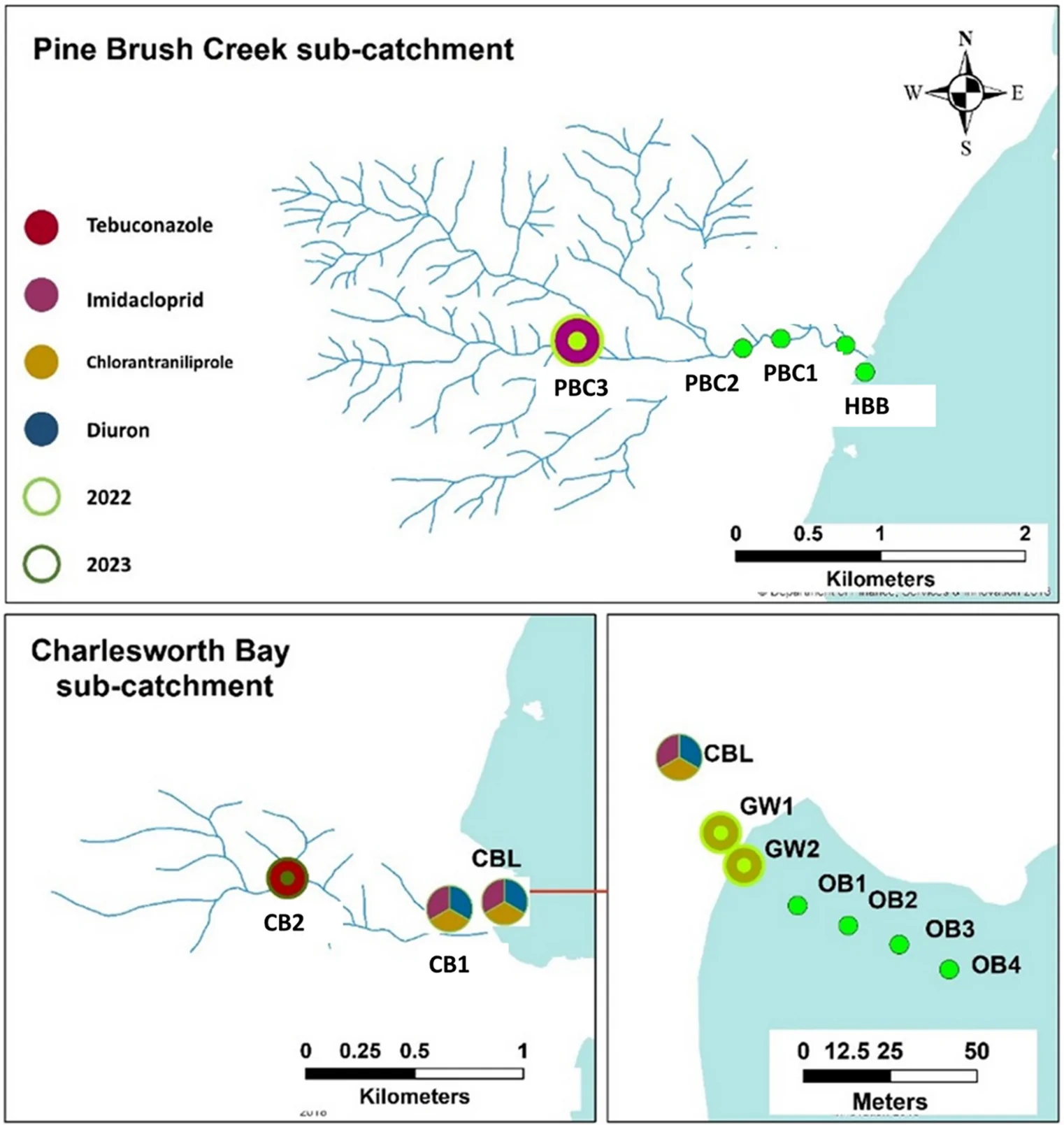
Pesticide occurrence in the Pine Brush Creek sub-catchment (**a**), Charlesworth Bay sub-catchment (catchment scale) (**b**) and Charlesworth Bay groundwater and ocean transect sites (**c**). Pesticide can be identified by symbols’ central colour and sampling year by symbol outline. Sites with no pesticide occurrence are marked in solid green

Pesticide contamination of aquatic ecosystems is a recognised issue of growing concern globally (Kumar et al. 2021; Mojiri et al. 2020). In line with our hypothesis that both surface and groundwater pathways contribute to contaminant delivery, pesticide occurrence displayed distinct temporal and hydrological patterns across the catchment. Four pesticides were identified over the research period: imidacloprid, diuron and chlorantraniliprole during the higher flow events in 2022, and tebuconazole during low flow periods in 2023 (Fig. 4). Pesticides were only detected in the lower catchment, suggesting nearby source(s) within the lower catchment such as inputs from the golf course, near CB1 site (Fig. 1). The identification of pesticides in groundwater should raise concerns due to the possible mobilisation in catchments impacted by high tidal groundwater exchange.

Imidacloprid was identified at the lower Charlesworth Bay sub-catchment site CB1 (0.07 µg L^−1^) and at Charlesworth Bay lagoon (0.02 µg L^−1^) in 2022. Imidacloprid was also detected just above the limit of detection (LOD, 0.01 µg L^−1^) at the upper Pine Brush Creek sub-catchment site PBC3 (0.02 µg L^−1^) during the same time period (Fig. 4). Imidacloprid is categorised as a neonicotinoid insecticide and is utilised to control a range of species including termites, aphids and thrips in urban and agricultural environments (Luo et al. 2021). Neonicotinoids are commonly utilised to treat seed stock due to broad spectrum effectiveness against a variety of pest species (Bandeira et al. 2020). Imidacloprid targets the nervous system, acting on nicotinic acetylcholine receptors (Stinson et al. 2022). The toxicity of imidacloprid on non-target species has been of growing concern worldwide (Bandeira et al. 2020; Thunnissen et al. 2022).

Chlorantraniliprole was identified at the limit of detection (0.1 µg L^−1^) at the lower Charlesworth Bay sub-catchment site CB1, as well as at the Charlesworth Bay lagoon (CBL), and within both groundwater wells (GW1 and GW2). Chlorantraniliprole is categorised as an anthranilic diamide insecticide, designed to control invertebrates from the class Lepidoptera, as well as a selection of Coleoptera species (Li et al. 2023; Stinson et al. 2022). Chlorantraniliprole operates via the activation of ryanodine receptors which stimulates the release of calcium, ultimately leading to paralysis and mortality (Khan et al. 2021; Li et al. 2023). Chlorantraniliprole was intended to specifically target invertebrates, with low operational efficiency on vertebrate species (Lahm et al. 2007; Stinson et al. 2022). However, recent studies indicate that the compound may activate the RYR1 receptor in mammals (Magyar et al. 2019; Truong and Pessah 2018).

Diuron (3-(3,4-Dichlorophenyl)−1,1-dimethylurea) was identified above the LOD (0.02 µg L^−1^) at the lower Charlesworth Bay sub-catchment site CB1 (0.13 µg L^−1^) and in the Charlesworth Bay lagoon (0.09 µg L^−1^) in 2022. Diuron has been found with growing frequency in aquatic ecosystems around the world (Hughes et al. 2018; Lambert et al. 2006; Nomura et al. 2023). Diuron targets the Hill reaction in the photosynthesis II system, inhibiting electron transfer and preventing formation of NADPH and ATP, required for essential biochemical processes in primary producers (Lambert et al. 2006; Silkina et al. 2009). The compound is predominantly used in agriculture but is also seen in urban and commercial settings for weed control (Kumar et al. 2010; Lambert et al. 2006; APVMA 2012). Diuron has also been used as an antifouling agent for ships (Kumar et al. 2010).

Diuron is persistent in sediment, with a predicted half-life of 175 days in lagoons (ANZECC 2000; Peterson and Batley 1991). Modelling of diuron in lagoon environments predicts 90% of diuron should occur within the sediment profile at our sites (Peterson and Batley 1991). Diuron adsorption onto sediment increases with rising temperatures and greater concentrations of organic matter (Peck et al. 1980). The significantly higher (*P* < 0.001) temperatures recorded at the CB1 site combined with the level of diuron recorded in the surface water raise concerns regarding potential diuron concentrations that may be found in lagoon sediments at this location. The occurrence of diuron at sites impacted by tidal groundwater exchange raises concerns regarding the potential mobilisation of this pesticide to sensitive marine ecosystems within the protected marine park.

Tebuconazole was identified just above the LOD (1 µg L^−1^) at the upper Charlesworth Bay sub-catchment site CB2 in 2023. Tebuconazole is classified as a conazole fungicide, a demethylation inhibitor suppressing germination and growth of fungal species (Vieira et al. 2022). Tebuconazole can induce toxicological stress on aquatic biota (Andreu‐Sánchez et al., 2012; Qi et al. 2015), impacting reproduction and development, as well as effects related to neurotoxicity and cellular function (Bošković et al. 2022). Tebuconazole also accelerates cyanobacterial blooms by inducing mortality of predatory species (Ortiz-Cañavate et al. 2019). Tebuconazole has high bioaccumulation potential in soil and sediment and can exhibit moderate solubility in surface water (Bošković et al. 2022). Tebuconazole can bind to topsoil in agricultural settings and subsequently transported to surrounding waterways in runoff events (Ortiz-Cañavate et al. 2019). The identification of the fungicide at the Charlesworth Bay upper sub-catchment site CB2 during low rainfall periods suggests tebuconazole is remobilising from the sediment profile in this system. Due to the inclusion of the research sites within the SIMP boundaries, guidelines for ecosystems with high conservation status due to ecological value should be applied. The level of protection for these sites recommends no change to chemical and physical stressors outside of natural variability, naturally occurring geologically linked contaminants should not exceed background concentrations, and the detection of anthropologically sourced contaminants, including pesticides, could be determined as grounds for source identification and subsequent management intervention (ANZECC 2000). Physical and chemical stressors include toxicants such as the heavy metals and pesticides found at the sites in this study (ANZECC 2000).

The identification of pesticides in groundwater and lagoon locations indicates potential mobilisation of contaminants via submarine groundwater discharge to the protected marine park. Pesticide occurrence in groundwater is of increasing concern in catchments around the world (Dugan et al. 2023). Patterns in pesticide contamination identified in this study were the same as the occurrence of pesticides identified in groundwater and surface water in Porto Alegre, Brazil, where the concentration of diuron and tebuconazole was found to be significantly higher in groundwater, while imidacloprid was linked to surface water mobilisation (Stefano et al. 2022).

## Conclusion

This study identified metal, pesticide, and nutrient contaminations in two sub-catchments located within developing coastal region on the East Coast of Australia, directly linked to a protected marine park. Total nitrogen and phosphate exceeded water quality guidelines during lower flow periods of 2023, indicating dilution during high flow periods. Copper exceeded environmental guidelines during rainfall events suggesting possible anthropogenic enrichment to the marine park. Zinc was found to be consistently above guidelines at all sites over both the 2022 and 2023 sampling regimes, possibly due to anthropogenic enrichment or naturally high geogenic levels in the region. Four pesticides were measured in sites immediately adjacent to a protected marine area. Overall, the results of this study suggest that rainfall, anthropogenic enrichment and/or geomorphological features are likely responsible for the mobilisation and transportation of enriched substance to the coastal ocean. The results of this research raise concern over potential toxicological implications for biota across protected marine parks directly linked to developing coastal regions.

## Data Availability

The datasets generated and/or analyzed during the current study are available from the corresponding author upon reasonable request.

## References

[CR1] Ahsanullah M, Williams A (1991) Sublethal effects and bioaccumulation of cadmium, chromium, copper and zinc in the marine amphipod Allorchestes compressa. Mar Biol 108:59-65

[CR2] Andreu‐Sánchez O, Paraíba LC, Jonsson CM, Carrasco JM (2012) Acute toxicity and bioconcentration of fungicide tebuconazole in zebrafish (*Danio rerio*). Environ Toxicol 27(2):109–116 21702075 10.1002/tox.20618

[CR3] ANZECC (2000) Australian and New Zealand guidelines for fresh and marine water quality. Australian and New Zealand Environment and Conservation Council and Agriculture and Resource Management Council of Australia and New Zealand, Canberra, vol 1, pp 1-314

[CR4] Atkinson CA, Jolley DF, Simpson SL (2007) Effect of overlying water pH, dissolved oxygen, salinity and sediment disturbances on metal release and sequestration from metal contaminated marine sediments. Chemosphere 69(9):1428–1437. 10.1016/j.chemosphere.2007.04.068 17568653

[CR5] Australian Pesticides and Veterinary Medicines Authority (APVMA) (2012) Diuron final review report: the reconsideration of the registrations of selected products containing diuron and their associated labels. https://www.apvma.gov.au/chemicals-and-products/chemical-review/listing/diuron

[CR6] Banaee M, Zeidi A, Mikušková N, Faggio C (2024) Assessing metal toxicity on crustaceans in aquatic ecosystems: a comprehensive review. Biol Trace Elem Res 202(12):5743–5761 10.1007/s12011-024-04122-7 38472509

[CR7] Bandeira FO, Lopes Alves PR, Hennig TB, Toniolo T, Natal-da-Luz T, Baretta D (2020) Effect of temperature on the toxicity of imidacloprid to *Eisenia andrei* and *Folsomia candida* in tropical soils. Environ Pollut 267. 10.1016/j.envpol.2020.115565 33254719

[CR8] Boretti A and Rosa L (2019) Reassessing the projections of the world water development report. NPJ Clean Water 2(1):15

[CR9] Bošković N, Bílková Z, Šudoma M, Bielská L, Škulcová L, Ribitsch D, Soja G, Vrana B, Hofman J (2022) Effects of biochar on the fate of conazole fungicides in soils and their bioavailability to earthworms and plants. Environ Sci Pollut Res 29(16):23323–23337 10.1007/s11356-021-17191-1 34807391

[CR10] Bourg ACM, Loch JG (1995) Mobilization of heavy metals as affected by pH and redox conditions. In: Biogeodynamics of pollutants in soils and sediments: risk assessment of delayed and non-linear responses. Berlin, Heidelberg: Springer Berlin Heidelberg, pp 87–102

[CR11] Cai Y, Cabrera JC, Georgiadis M, Jayachandran K (2002) Assessment of arsenic mobility in the soils of some golf courses in South Florida. Sci Total Environ 291(1):123–134. 10.1016/S0048-9697(01)01081-6 12150432

[CR12] Champion DC, Brown C, Mathews E, Huston DL, Kositcin N (2016) Geodynamic synthesis of the Phanerozoic of eastern Australia (Record 2016/12). Geoscience Australia

[CR13] Chuan M, Shu G, Liu J (1996) Solubility of heavy metals in a contaminated soil: effects of redox potential and pH. Water, Air, Soil Pollut 90:543-556

[CR14] Conrad SR, Santos IR, White S, Sanders CJ (2019) Nutrient and trace metal fluxes into estuarine sediments linked to historical and expanding agricultural activity (Hearnes Lake, Australia). Estuaries Coasts 42(4):944–957. 10.1007/s12237-019-00541-1

[CR15] Conrad SR, Santos IR, White SA, Woodrow RL, Sanders CJ (2021) Cryptic night-time trace metal and metalloid contamination in an intensively cultivated coastal catchment. Environ Pollut 276. 10.1016/j.envpol.2021.116685 33636558

[CR16] DeLorenzo ME, Scott GI, Ross PE (2001) Toxicity of pesticides to aquatic microorganisms: a review. Environ Toxicol Chem: An Int J 20(1):84-98 10.1897/1551-5028(2001)020<0084:toptam>2.0.co;2 11351418

[CR17] Dobriyal P, Badola R, Tuboi C, Hussain SA (2017) A review of methods for monitoring streamflow for sustainable water resource management Appl Water Sci 7(6):2617-2628. 10.1007/s13201-016-0488-y

[CR18] Dugan ST, Muhammetoglu A, Uslu A (2023) A combined approach for the estimation of groundwater leaching potential and environmental impacts of pesticides for agricultural lands. Sci Total Environ 901:16589 https://ro.uow.edu.au/scipapers/978 10.1016/j.scitotenv.2023.165892 37524174

[CR19] Fergusson CL (2010) Interpreting New England subduction complex rocks using deep-sea drilling results from the Nankai Trough (off shore southwest Japan). University of Wollongong Research Online, pp 149–155. Retrieved January 18, 2025, from https://hdl.handle.net/10779/uow.27777057

[CR20] Franklin N, Adams M, Stauber J, Lim R (2001) Development of an improved rapid enzyme inhibition bioassay with marine and freshwater microalgae using flow cytometry. Arch Environ Contam Toxicol 40:469–480 10.1007/s002440010199 11525489

[CR21] Geoscience Australia (2023) National hydrogeological inventory. Canberra: Geoscience Australia. 10.26186/148884

[CR22] Gilligan LB, Brownlow JW, Cameron RG, Henley HF (1992) Dorrigo–Coffs Harbour 1:250,000 metallogenic map SH/56-10, SH/56-11: metallogenic study and mineral deposit data sheets. Geological Survey of New South Wales. https://search.geoscience.nsw.gov.au/product/1552

[CR23] Giménez-Forcada E, Vega-Alegre M (2015) Arsenic, barium, strontium and uranium geochemistry and their utility as tracers to characterize groundwaters from the Espadán-Calderona Triassic Domain, Spain. Sci Total Environ 512:599–612 10.1016/j.scitotenv.2014.12.010 25655986

[CR24] Gomez-Alvarez P, Bates B, Santos IR, Escobar Correa R, Tucker JP, Ibrahim N, Laicher-Edwards D, Gardner K, Silva Monteiro L, Silva DA (2019) Submarine groundwater discharge revealed by ^224^Ra and ^223^Ra in Coffs Harbour, Australia. J Radioanal Nucl Chem 319:1193–1199

[CR25] Gopalakrishnan S, Thilagam H, Raja PV (2008) Comparison of heavy metal toxicity in life stages (spermiotoxicity, egg toxicity, embryotoxicity and larval toxicity) of *Hydroides elegans*. Chemosphere 71(3):515–528 18022210 10.1016/j.chemosphere.2007.09.062

[CR26] Gutiérrez-Martín D, Gil-Solsona R, Saaltink MW, Rodellas V, López-Serna R, Folch A, Carrera J, Gago-Ferrero P (2023) Chemicals of emerging concern in coastal aquifers: assessment along the land-ocean interface. J Hazard Mater 448:130876 10.1016/j.jhazmat.2023.130876 36736215

[CR27] Handy R (1994) Intermittent exposure to aquatic pollutants: assessment, toxicity and sublethal responses in fish and invertebrates. Comp Biochem Physiol C Pharmacol Toxicol Endocrinol 107(2):171–184

[CR28] Hao X, Ouyang W, Gu X, He M, Lin C (2024) Accelerated export and transportation of heavy metals in watersheds under high geological backgrounds. J Hazard Mater 465. 10.1016/j.jhazmat.2024.133514 38228005

[CR29] Harley J, Carvalho L, Dudley B, Heal K, Rees R, Skiba U (2015) Spatial and seasonal fluxes of the greenhouse gases N_2_O, CO_2_ and CH_4_ in a UK macrotidal estuary. Estuar Coast Shelf Sci 153:62–73

[CR30] Henriques B, Rocha LS, Lopes CB, Figueira P, Monteiro RJR, Duarte AC, Pardal MA, Pereira E (2015) Study on bioaccumulation and biosorption of mercury by living marine macroalgae: prospecting for a new remediation biotechnology applied to saline waters. Chem Eng J 281:759–770. 10.1016/j.cej.2015.07.013

[CR31] Hooten RL, Carr RS (1998) Development and application of a marine sediment pore‐water toxicity test using Ulva fasciata zoospores. Environ Toxicol Chem: An Int J 17(5):932-940

[CR32] Howe PL, Reichelt-Brushett AJ, Clark MW (2014) Effects of Cd, Co, Cu, Ni and Zn on asexual reproduction and early development of the tropical sea anemone Aiptasia pulchella. Ecotoxicology 23:1593-1606 10.1007/s10646-014-1299-2 25119449

[CR33] Hughes RG, Potouroglou M, Ziauddin Z, Nicholls JC (2018) Seagrass wasting disease: nitrate enrichment and exposure to a herbicide (Diuron) increases susceptibility of *Zostera marina* to infection. Mar Pollut Bull 134:94–98. 10.1016/j.marpolbul.2017.08.032 28844456

[CR34] Isbell R (2016) The Australian soil classification. Commonwealth Scientific and Industry Research Organisation Publishing

[CR35] Ismanto A, Hadibarata T, Widada S, Indrayanti E, Ismunarti DH, Safinatunnajah N, Kusumastuti W, Dwiningsih Y, Alkahtani J (2023) Groundwater contamination status in Malaysia: level of heavy metal, source, health impact, and remediation technologies. Bioprocess Biosyst Eng 46(3):467-482 10.1007/s00449-022-02826-5 36520279

[CR36] Jessop K, Daczko N, Piazolo S (2019) Tectonic cycles of the New England Orogen, eastern Australia: a review. Aust J Earth Sci 66:1-38. 10.1080/08120099.2018.1548378

[CR37] Kapoor D, Singh MP (2021) Heavy metal contamination in water and its possible sources. Heavy metals in the environment. Elsevier, pp 179–189

[CR38] Keimowitz AR, Zheng Y, Chillrud SN, Mailloux B, Jung HB, Stute M, Simpson HJ (2005) Arsenic redistribution between sediments and water near a highly contaminated source. Environ Sci Technol 39(22):8606-8613 10.1021/es050727t 16329197

[CR39] Khan MM, Hafeez M, Elgizawy K, Wang H, Zhao J, Cai W, Ma W, Hua H (2021) Sublethal effects of chlorantraniliprole on Paederus fuscipes (Staphylinidae: Coleoptera), a general predator in paddle field. Environ Pollut 291:118171 10.1016/j.envpol.2021.118171 34562692

[CR40] Kumar KS, Choo K-s, Yea SS, Seo Y, Han T (2010) Effects of the phenylurea herbicide diuron on the physiology of *Saccharina japonica* Aresch. Toxicol Environ Health Sci 2(3):188–199. 10.1007/BF03216505

[CR41] Kumar R, Sankhla MS, Kumar R, Sonone SS (2021) Impact of pesticide toxicity in aquatic environment. Biointerfaces Res Appl Chem 11(3):10131-10140

[CR42] Laffont-Schwob I, Triboit F, Prudent P, Soulié-Märsche I, Rabier J, Despréaux M, Thiéry A (2015) Trace metal extraction and biomass production by spontaneous vegetation in temporary Mediterranean stormwater highway retention ponds: freshwater macroalgae (*Chara* spp.) vs. cattails (*Typha* spp.). Ecol Eng 81:173–181. 10.1016/j.ecoleng.2015.04.052

[CR43] Lahm GP, Stevenson TM, Selby TP, Freudenberger JH, Cordova D, Flexner L, Bellin CA, Dubas CM, Smith BK, Hughes KA (2007) Rynaxypyr™: a new insecticidal anthranilic diamide that acts as a potent and selective ryanodine receptor activator. Bioorg Med Chem Lett 17(22):6274–6279 10.1016/j.bmcl.2007.09.012 17884492

[CR44] Laicher D, Benkendorff K, White S, Conrad S, Woodrow RL, Butcherine P, Sanders CJ (2022) Pesticide occurrence in an agriculturally intensive and ecologically important coastal aquatic system in Australia. Mar Pollut Bull 180. 10.1016/j.marpolbul.2022.113675 35642798

[CR45] Lambert SJ, Thomas KV, Davy AJ (2006) Assessment of the risk posed by the antifouling booster biocides Irgarol 1051 and diuron to freshwater macrophytes. Chemosphere 63(5):734–743. 10.1016/j.chemosphere.2005.08.023 16213569

[CR46] Li Z, Sheng Y, Yang J, Burton ED (2016) Phosphorus release from coastal sediments: impacts of the oxidation-reduction potential and sulfide. Mar Pollut Bull 113(1):176–181. 10.1016/j.marpolbul.2016.09.007 27614565

[CR47] Li C, Quan Q, Gan Y, Dong J, Fang J, Wang L, Liu J (2020) Effects of heavy metals on microbial communities in sediments and establishment of bioindicators based on microbial taxa and function for environmental monitoring and management. Sci Total Environ 749. 10.1016/j.scitotenv.2020.141555 32841857

[CR48] Li X, Tu M, Yang B, Zhang Q, Li H, Ma W (2023) Chlorantraniliprole in foods: determination, dissipation and decontamination. Food Chem 406. 10.1016/j.foodchem.2022.135030 36446283

[CR49] Li L, Barry DA, Jeng DS, Prommer H (2004) Tidal dynamics of groundwater flow and contaminant transport in coastal aquifers. Coast Aquifer Management: Monitoring, modeling, and case studies, pp 115-141

[CR50] Liao W, Zhu Z, Feng C, Yan Z, Hong Y, Liu D, Jin X (2023) Toxicity mechanisms and bioavailability of copper to fish based on an adverse outcome pathway analysis. J Environ Sci (China) 127:495–507. 10.1016/j.jes.2022.06.002 36522080

[CR51] Linklater M, Ingleton TC, Kinsela MA, Morris BD, Allen KM, Sutherland MD, Hanslow DJ (2019) Techniques for classifying seabed morphology and composition on a subtropical-temperate continental shelf. Geosciences 9(3):141. 10.3390/geosciences9030141

[CR52] Liu J-J, Diao Z-H, Xu X-R, Xie Q (2019) Effects of dissolved oxygen, salinity, nitrogen and phosphorus on the release of heavy metals from coastal sediments. Sci Total Environ 666:894–901. 10.1016/j.scitotenv.2019.02.288 30818213

[CR53] Lu Y, Yuan J, Lu X, Su C, Zhang Y, Wang C, Cao X, Li Q, Su J, Ittekkot V (2018) Major threats of pollution and climate change to global coastal ecosystems and enhanced management for sustainability. Environ Pollut 239:670-680 10.1016/j.envpol.2018.04.016 29709838

[CR54] Luo T, Wang X, Jin Y (2021) Low concentrations of imidacloprid exposure induced gut toxicity in adult zebrafish (*Danio rerio*). Comp Biochem Physiol C Toxicol Pharmacol 241. 10.1016/j.cbpc.2020.108972 33418081

[CR55] Luo M, Zhang Y, Li H, Hu W, Xiao K, Yu S, Zheng C, Wang X (2022) Pollution assessment and sources of dissolved heavy metals in coastal water of a highly urbanized coastal area: the role of groundwater discharge. Sci Total Environ 807:151070 10.1016/j.scitotenv.2021.151070 34699837

[CR56] Magyar ZÉ, Diszházi G, Péli-Szabó J, Szentesi P, Collet C, Csernoch L, Nánási P, Almássy J (2019) The diamide insecticide chlorantraniliprole increases the single-channel current activity of the mammalian skeletal muscle ryanodine receptor. Gen. Physiol. Biophys 38(183–186):30821253 10.4149/gpb_2019007 30821253

[CR57] Malcolm HA, Davies PL, Jordan A, Smith SDA (2011) Variation in sea temperature and the East Australian Current in the Solitary Islands region between 2001–2008. Deep Sea Res Part II Top Stud Oceanogr 58(5):616–627. 10.1016/j.dsr2.2010.09.030

[CR58] Marandi A, Karro E, Puura E (2004) Barium anomaly in the Cambrian-Vendian aquifer system in North Estonia. Environ Geol 47:132–139

[CR59] Marcovecchio JE, Gerpe MS, Bastida RO, Rodríguez DH, Morón S (1994) Environmental contamination and marine mammals in coastal waters from Argentina: an overview. Sci Total Environ 154(2-3):141-151 10.1016/0048-9697(94)90084-1 7973603

[CR60] Martin M, Osborn KE, Billig P, Glickstein N.(1981) Toxicities of ten metals to Crassostrea gigas and Mytilus edulis embryos and Cancer magister larvae. Mar Pollut Bull 12(9):305-308

[CR61] McKnight KS, Gissi F, Adams MS, Stone S, Jolley D, Stauber J (2023) The effects of nickel and copper on tropical marine and freshwater microalgae using single and multispecies tests. Environ Toxicol Chem 42(4):901-913 10.1002/etc.5565 36896707

[CR62] Michalak I, Messyasz B (2021) Concise review of Cladophora spp.: macroalgae of commercial interest. J Appl Phycol 33(1):133-166

[CR63] Middag R, Van Hulten M, Van Aken H, Rijkenberg M, Gerringa L, Laan P, De Baar H (2015) Dissolved aluminium in the ocean conveyor of the West Atlantic Ocean: effects of the biological cycle, scavenging, sediment resuspension and hydrography. Mar Chem 177:69-86

[CR64] Mojiri A, Zhou JL, Robinson B, Ohashi A, Ozaki N, Kindaichi T, Farraji H, Vakili M (2020) Pesticides in aquatic environments and their removal by adsorption methods. Chemosphere 253:126646 10.1016/j.chemosphere.2020.126646 32276120

[CR65] Mokoena P, Manyama K, van Bever Donker J, Kanyerere T (2021) Investigation of groundwater salinity using geophysical and geochemical approaches: Heuningnes catchment coastal aquifer. Western Cape Province, South Africa. Environ Earth Sci 80(5)

[CR66] Moody DW (1990) Groundwater contamination in the United States. J Soil Water Conserv 45(2):170-179

[CR67] Moore WS (1997) High fluxes of radium and barium from the mouth of the Ganges-Brahmaputra River during low river discharge suggest a large groundwater source. Earth Planet Sci Lett 150(1-2):141-150

[CR68] Moreira VA, Cravo-Laureau C, de Carvalho ACB, Baldy A, Bidone ED, Sabadini-Santos E, Duran R (2023) Microbial indicators along a metallic contamination gradient in tropical coastal sediments. J Hazard Mater 443:130244. 10.1016/j.jhazmat.2022.130244 36327839

[CR69] Morrissy JG, Currell MJ, Reichman SM, Surapaneni A, Megharaj M, Crosbie ND, Hirth D, Aquilina S, Rajendram W, Ball AS (2021) Nitrogen contamination and bioremediation in groundwater and the environment: a review. Earth-Sci Rev 222:103816

[CR70] Nadella SR, Fitzpatrick JL, Franklin N, Bucking C, Smith S, Wood CM (2009) Toxicity of dissolved Cu, Zn, Ni and Cd to developing embryos of the blue mussel (Mytilus trossolus) and the protective effect of dissolved organic carbon. Comp Biochem Physiol Part C: Toxicol Pharm 149(3):340-348 10.1016/j.cbpc.2008.09.001 18832046

[CR71] Nomura M, Okamura H, Horie Y, Yap CK, Emmanouil C, Uwai S, Kawai H (2023) Effects of antifouling compounds on the growth of macroalgae *Undaria pinnatifida*. Chemosphere 312. 10.1016/j.chemosphere.2022.137141 36343734

[CR72] Nordstrom DK (2011) Hydrogeochemical processes governing the origin, transport and fate of major and trace elements from mine wastes and mineralized rock to surface waters. Appl Geochem 26(11):1777-1791

[CR73] Noulas C, Tziouvalekas M, Karyotis T (2018) Zinc in soils, water and food crops. J Trace Elem Med Biol 49:252-260. 10.1016/j.jtemb.2018.02.009 29472130

[CR74] O’Day PA, Vlassopoulos D, Root R, Rivera N (2004) The influence of sulfur and iron on dissolved arsenic concentrations in the shallow subsurface under changing redox conditions. Proc Nat Acad Sci 101(38):13703-13708 10.1073/pnas.0402775101 PMC518762 15356340

[CR75] Obiora SC, Chukwu A, Davies TC (2019) Contamination of the potable water supply in the lead–zinc mining communities of Enyigba, Southeastern Nigeria. Mine Water Environ 38(1):148-157

[CR76] Ortiz-Cañavate BK, Wolinska J, Agha R (2019) Fungicides at environmentally relevant concentrations can promote the proliferation of toxic bloom-forming cyanobacteria by inhibiting natural fungal parasite epidemics. Chemosphere 229:18-21 10.1016/j.chemosphere.2019.04.203 31063876

[CR77] Peck DE, Corwin DL, Farmer WJ (1980) Adsorption‐desorption of diuron by freshwater sediments, vol 9, no. 1. American Society of Agronomy, Crop Science Society of America, and Soil Science Society of America, pp 101–106

[CR78] Peterson SM, Batley GE (1991) Fate and transport of endosulfan and diuron in aquatic ecosystems. CSIRO, Centre for Advanced Analytical Chemistry

[CR79] Poor CJ, McDonnell JJ (2007) The effects of land use on stream nitrate dynamics. J Hydrol 332(1-2):54-68

[CR80] Power J, Schepers J (1989) Nitrate contamination of groundwater in North America. Agric Ecosyst Environ 26(3-4):165-187

[CR81] Prieto C, Destouni G (2005) Quantifying hydrological and tidal influences on groundwater discharges into coastal waters. Water Resour Res 41(12)

[CR82] Qi SZ, Chen XF, Liu Y, Jiang JZ, Wang CJ (2015) Comparative toxicity of rac-and S-tebuconazole to Daphnia magna. J Environ Sci Health, Part B, 50(7):456-462 10.1080/03601234.2015.1018756 25996809

[CR83] Qin Y, Zhang F, Xue S, Ma T, Yu,L (2022) Heavy metal pollution and source contributions in agricultural soils developed from Karst Landform in the Southwestern Region of China. Toxics 10(10). 10.3390/toxics10100568 PMC9610029 36287848

[CR84] Reading MJ, Santos IR, Maher DT, Jeffrey LC, Tait DR (2017) Shifting nitrous oxide source/sink behaviour in a subtropical estuary revealed by automated time series observations. Estuar Coast Shelf Sci 194:66-76. 10.1016/j.ecss.2017.05.017

[CR85] Real MKH, Varol M, Rahman MS, Islam ARMT (2024) Pollution status and ecological risks of metals in surface water of a coastal estuary and health risk assessment for recreational users. Chemosphere 348. 10.1016/j.chemosphere.2023.140768 38000553

[CR86] Reijnders PJ, Aguilar A, Borrell A (2009) Pollution and marine mammals. Encyclopedia of marine mammals. Elsevier, pp 890–898

[CR87] Rubalingeswari N, Thulasimala D, Giridharan L, Gopal V, Magesh N, Jayaprakash M (2021) Bioaccumulation of heavy metals in water, sediment, and tissues of major fisheries from Adyar estuary, Southeast coast of India: an ecotoxicological impact of a metropolitan city. Mar Pollut Bull 163:111964 10.1016/j.marpolbul.2020.111964 33450441

[CR88] Rubinos DA, Iglesias L, Díaz-Fierros F, Barral MT (2011) Interacting effect of pH, phosphate and time on the release of arsenic from polluted river sediments (Anllóns River, Spain). Aquatic Geochemistry 17(3):281–306. 10.1007/s10498-011-9135-2

[CR89] Rybak A, Messyasz B, Łęska B (2012) Freshwater *Ulva* (Chlorophyta) as a bioaccumulator of selected heavy metals (Cd, Ni and Pb) and alkaline earth metals (Ca and Mg). Chemosphere 89(9):1066–1076. 10.1016/j.chemosphere.2012.05.071 22726424

[CR90] Santos IR, Chen X, Lecher AL, Sawyer AH, Moosdorf N, Rodellas V, Tamborski J, Cho HM, Dimova N, Sugimoto R (2021) Submarine groundwater discharge impacts on coastal nutrient biogeochemistry. Nat Rev Earth Environ 2(5):307-323

[CR91] Sheng D, Meng X, Wen X, Wu J, Yu H, Wu M, Zhou T (2023) Hydrochemical characteristics, quality and health risk assessment of nitrate enriched coastal groundwater in northern China. J Clean Prod 403

[CR92] Silkina A, Bazes A, Vouvé F, Le Tilly V, Douzenel P, Mouget J-L, Bourgougnon N (2009) Antifouling activity of macroalgal extracts on *Fragilaria pinnata* (Bacillariophyceae): a comparison with Diuron. Aquat Toxicol 94(4):245–254. 10.1016/j.aquatox.2009.07.004 19726092

[CR93] Smith K, Scarpaci C, Scarr MJ, Otway N (2014) Scuba diving tourism with critically endangered grey nurse sharks (Carcharias taurus) off eastern Australia: tourist demographics, shark behaviour and diver compliance. Tour Manag 45:211-225

[CR94] Stefano PHP, Roisenberg A, Santos MR, Dias MA, Montagner CC (2022) Unraveling the occurrence of contaminants of emerging concern in groundwater from urban setting: a combined multidisciplinary approach and self-organizing maps. Chemosphere 299. 10.1016/j.chemosphere.2022.134395 35339518

[CR95] Stinson SA, Hasenbein S, Connon RE, Deng X, Alejo JS, Lawler SP, Holland EB (2022) Agricultural surface water, imidacloprid, and chlorantraniliprole result in altered gene expression and receptor activation in Pimephales promelas. Sci Total Environ 806:150920 10.1016/j.scitotenv.2021.150920 PMC8892843 34653458

[CR96] Tait DR, Santos IR, Lamontagne S, Sippo JZ, McMahon A, Jeffrey LC, Maher DT (2023) Submarine groundwater discharge exceeds river inputs as a source of nutrients to the Great Barrier Reef. Environ Sci Technol 57(41):15627-15634 10.1021/acs.est.3c03725 37805932

[CR97] Thunnissen NW, Geurts KAG, Hoeks S, Hendriks AJ (2022) The impact of imidacloprid and thiacloprid on the mean species abundance in aquatic ecosystems. Sci Total Environ 822. 10.1016/j.scitotenv.2022.153626 35124047

[CR98] Truong KM, Pessah IN (2018) Comparison of chlorantraniliprole and flubendiamide activity toward wild-type and malignant hyperthermia-susceptible ryanodine receptors and heat stress intolerance. Toxicol Sci 167:509–523 10.1093/toxsci/kfy256 PMC6358238 30329129

[CR99] Vieira RS, Venâncio CA, Félix LM (2022) Behavioural impairment and oxidative stress by acute exposure of zebrafish to a commercial formulation of tebuconazole. Environ Toxicol Pharmacol 91:103823 10.1016/j.etap.2022.103823 35123019

[CR100] Wang N, Ye Z, Huang L, Zhang C, Guo Y, Zhang W (2022) Arsenic occurrence and cycling in the aquatic environment: a comparison between freshwater and seawater. Water 15(1):147

[CR101] Wang Z, Wang Q, Guo Y, Yu S, Xiao K, Zhang Y, Li H, Zheng C, Geng X, Zhang X (2023) Seawater–groundwater interaction governs trace metal zonation in a coastal sandy aquifer. Water Res Res 59(9):e2022WR032828

[CR102] Wadnerkar PD, Santos IR, Looman A, Sanders CJ, White S, Tucker JP, Holloway C (2019) Significant nitrate attenuation in a mangrove-fringed estuary during a flood-chase experiment. Environ Pollut 253:1000–1008. 10.1016/j.envpol.2019.06.060 31434177

[CR103] Werner AD, Lockington DA (2006) Tidal impacts on riparian salinities near estuaries. J Hydrol 328(3-4):511-522

[CR104] Woodwell GM, Rich PH, Hall CAS (1973) Carbon in estuaries. In: Brookhaven Symposia in Biology, Vol. 24 pp. 221-240 4807338

[CR105] Yee N, Finley L, Roberts A, Kelaher B, Millar A (2019) Re-discovery of the critically endangered marine brown alga, Nereia lophocladia (Order Sporochnales) at Coffs Harbour, NSW, Australia. Mar Biodivers 49:527-529

[CR106] Yin H, Niu J, Ren Y, Cong J, Zhang X, Fan F, Xiao Y, Zhang X, Deng J, Xie M, He Z, Zhou J, Liang Y, Liu X (2015) An integrated insight into the response of sedimentary microbial communities to heavy metal contamination. Sci Rep 5(1). 10.1038/srep14266 26391875 PMC4585741

[CR107] YSI Environmental (2008) ORP Management in wastewater as an indicator of process efficiency. YSI, Yellow Springs, OH. https://www.ysi.com/ysi-blog/water-blogged-blog/2013/08/orp-management-in-wastewater-as-an-indicator-of-process-efficiency?srsltid=AfmBOor3qYH6PbvdwSOm6hLFORF177yTFbZ1xS8IPq8WK7VPBSYucHEG

[CR108] Zhang Q, Volker RE, Lockington DA (2002) Experimental investigation of contaminant transport in coastal groundwater. Adv Environ Res 6(3):229-237

[CR109] Zhao Y, Zhang Z, Wang G, Li X, Ma J, Chen S, Deng H, Annalisa O-H (2019) High sulfide production induced by algae decomposition and its potential stimulation to phosphorus mobility in sediment. Sci Total Environ 650:163–172. 10.1016/j.scitotenv.2018.09.010 30196216

